# Clinicopathological and molecular characterization of astroblastoma

**DOI:** 10.3389/fnmol.2025.1483833

**Published:** 2025-02-03

**Authors:** Xiaoyan Wu, Wenfeng Peng, Xu Zhang, Tao Tang, Ling Deng, Yuxia Xu, Xiaoyun Liu, Fang Wang, Wujian Peng, Jianrong Huang, Xiaoni Zhong

**Affiliations:** ^1^Department of Molecular Diagnostics, State Key Laboratory of Oncology in South China, Guangdong Provincial Clinical Research Center for Cancer, Sun Yat-sen University Cancer Center, Guangzhou, China; ^2^Department of Pathology, Shenzhen Second People’s Hospital, Shenzhen University 1st Affiliated Hospital, Shenzhen University School of Medicine, Shenzhen, China; ^3^Department of Nephrology, The Third People’s Hospital of Shenzhen, The Second Affiliated Hospital of Southern University of Science and Technology, Shenzhen, China; ^4^Department of Pathology, Shenzhen People’s Hospital, The Second Affiliated Hospital of Jinan University, The First Affiliated Hospital of Southern University of Science and Technology, Shenzhen, China

**Keywords:** astroblastoma, clinicopathological, molecular characterization, next-generation sequencing, EWSR1-NUDT10 gene fusion

## Abstract

**Introduction:**

Astroblastoma is a rare tumour of the central nervous system that often manifests with non-specific clinical symptoms and lacks distinct histological features. There is a pressing need for further understanding of the clinicopathological and molecular characteristics of Astroblastoma. Identifying mutant genes can aid in reliable and early diagnosis, as well as provide insights for the development of targeted therapies.

**Methods:**

This study aims to investigate the clinicopathologic and molecular characteristics of astroblastoma. A total of four patients diagnosed with astroblastoma were included in the analysis. Clinical features, histological findings, and immunohistochemistry results were reviewed and analyzed. Genetic alterations were identified using fluorescence *in situ* hybridization (FISH) and next-generation sequencing (NGS), followed by patient follow-up.

**Results:**

The study included four female patients, ranging in age from 8 to 44 years. One patient had a tumour in the right parietal lobe, while the other three had tumours in the spinal cord. Histology is usually characterized by pseudorosettes of astroblasts and hyalinization of blood vessels. These tumors showed a growth pattern similar to traditional intracranial astroblastoma, and the histological manifestations of the four patients were all high-grade, showing features of high-density areas of tumor cells or necrosis. Immunohistochemical staining revealed that all four patients expressed OLIG2, EMA, and vimentin, while three patients also expressed GFAP and S-100. The Ki-67 positivity index was approximately 15% in three cases and 10% in one case. Fluorescence *in situ* hybridization (FISH) using break-apart probes showed EWRS1 breaks in three patients and MN1 breaks in one. Further DNA or RNA-targeted biallelic sequencing identified an EWSR1(Exon1-7)-BEND2(Exon2-14) fusion in case 1, and an EWSR1(Exon1-7)-BEND2(Intergenic) fusion in case 2. In case 3, an EWSR1(Exon1-7)-NUDT10(Intergenic) fusion was present, and in case 4, an MN1(Exon1)-BEND2(Exon2) fusion was identified. The EWSR1-NUDT10 gene fusion is a new fusion type in astroblastoma. The patients were followed up for 76.5, 17.6, 33.7, and 61.3 months, respectively. Three cases experienced tumour recurrences at the spinal cord site, with multiple recurrences in case 4.

**Discussion:**

Our study unveiled the distinctive clinicopathological and molecular mutational characteristics of astroblastoma, while also identifying rare mutated genes. Additionally, the detection of MN1 or EWSR1 gene fusion through FISH or next-generation sequencing can provide valuable insights into the molecular mechanisms and aid in the differential diagnosis of astroblastoma.

## Introduction

Astroblastoma is a rare neuroepithelial tumour, accounting for only 0.45 to 2.8% of intracranial primary gliomas. This type of tumour is more frequently observed in children and young adults, with a higher prevalence among female (Hirose et al., 2018; Lehman et al., 2022). First discovered and reported by Bailev and Bucy (2007) in 1926, astroblastoma was initially classified as a neuroepithelial tumour of undetermined origin and tentatively ungraded. It was later considered as a limited glioma under the name “astroblastoma, MN1 variant” in the 5th edition of the WHO classification of tumours of the central nervous system. The MN1 gene is located on 22q11 and encodes a protein of more than 1,300 amino acids, but most of it is an intrinsically disordered region (IDR) and lacks other secondary structures or known domains. Therefore, MN1 can only serve as a transcriptional regulatory cofactor, playing a role in transcriptional regulation by combining with other transcription factors. Astroblastomas are known to be associated with MN1 gene alterations, with common occurrences of MN1-BEND2 and MN1-CXXC5 gene fusions. CXXC5 (CXXC Finger Protein 5) is a member of the CXXC protein family that specifically recognizes and binds to unmethylated DNA CpG island regions. Within the Wnt/β-catenin signaling pathway, CXXC5 serves as a negative regulator by directly interacting with β-catenin and inhibiting its transcriptional activity. This inhibition subsequently affects various processes such as cell proliferation, differentiation, and migration that are dependent on the Wnt signaling pathway. Recent reports have identified individuals with astroblastomas carrying the EWSR1-BEND2 fusion (Yamasaki et al., 2020). The EWSR1 gene is situated on chromosome 22q12 and belongs to the TET family, encoding an RNA-binding protein (Alhalabi et al., 2021). It comprises 17 coding exons, which produce a nuclear protein consisting of 656 amino acids, and is implicated in the onset and progression of various tumors (Jo, 2020; Agaimy et al., 2023). The N-terminal activation domain of EWSR1 has been shown to fuse with the DNA-binding domains of multiple transcription factors, leading to the production of oncogenic transcription factors (Fernandes Arroteia et al., 2024). BEND2 (Xp22.13) encodes one of nine human proteins that contain BEN domains and features two tandem BEN domains at its C terminus. These BEN domains are predicted to facilitate protein-DNA interactions and chromatin remodeling (Lehman, 2023). In certain proteins, BEN domains have been shown to bind directly to DNA in a sequence-specific manner (Dai et al., 2013; Nakayama et al., 2020). This suggests that BEND2 plays a significant role in tumorigenesis and development. Molecular stratification diagnosis is crucial for enhancing the accuracy of distinguishing between different types of astroblastoma.

Next-generation sequencing assays can analyze the complete coding region of a gene, enabling the identification of both known and novel variants within the gene. The advancement and refinement of next-generation sequencing technology have significantly enhanced our ability to delve into the molecular mechanisms underlying the occurrence, progression, and prognosis of astroblastoma. The widespread adoption of next-generation sequencing has led to the identification of a growing number of rare mutations specific to astroblastomas. Despite this progress, there remains a scarcity of research focused on astroblastoma mutations using next-generation sequencing methods, with limited literature available both domestically and internationally on this particular tumour.

In this study, we collected four cases of rare astroblastoma from the Department of Pathology at Shenzhen Second People’s Hospital and the Department of Molecular Diagnostics at Sun Yat-sen University Affiliated Cancer Hospital. Our aim was to analyze the clinical manifestations and characteristics of different molecular subtypes of astroblastoma. We conducted a retrospective study of histology and immunohistochemistry, focusing on MN1 fusion-positive astroblastoma (MN1-BEND2 fusion), EWSR1 fusion-positive astroblastoma (EWSR1-BEND2 fusion), and other subtypes. This work seeks to enhance the understanding of this tumor.

## Materials and methods

### Tumor samples

Four cases of astroblastoma confirmed by the Department of Pathology of the Shenzhen Second People’s Hospital and the Sun Yat-sen University Cancer Center from March 2018 to May 2024 were collected. The classification of the samples was based on the 5th edition of the WHO Classification of Tumors of the Central Nervous System. Clinical, pathophysiologic, immunophenotypic, genetic testing and follow-up data were assessed through an electronic case system. The study was conducted with approval from the Ethics Committee of Sun Yat-sen University Cancer Prevention and Control Center (B2020-344-01) and in accordance with the Declaration of Helsinki.

### Hematoxylin and eosin (H&E) and immunohistochemical (IHC) stains

The postoperative specimens were fixed in 10% neutralized formalin, dehydrated conventionally, embedded in paraffin, sectioned at a thickness of 3 μm, and stained with HE. Immunohistochemical staining was carried out using an indirect detection method with enzyme-labelled Multimer multimers on a Benchmark Ultra automated immunostainer (Ventana, Tucson, AZ). Negative and positive controls were included, and the primary antibody reagents used were GFAP, S-100, OLIG2, EMA, vimentin, and Ki-67 (refer to Table 1). The staining procedure was as follows: (1) label the 3 μm paraffin section slide with the name of the primary antibody and bake the slide at 75°C for 60 min. (2) Enter the name of each primary antibody into the computer system, and the Ebar barcode printer will print the label, which should then be affixed to each slide. (3) Verify that there are sufficient reagents in each machine to complete the experiment. (4) Wipe dry any moisture on the back of the slices and the surface of the label, then place it on the slicing plate. (5) Position the selected antibodies at the designated spots on the machine according to the experimental requirements, and then place the DAB kit, hematoxylin, blue return solution, and other reagent bottles on the detection reagent rack. (6) Start the computer and run the program. (7) After staining is completed, open the sectioning tray and remove the sections. Begin with adding detergent to clean off any oil from the slides, then proceed with routine dehydration, transparency, and sealing. Immunohistochemical staining reveals brown granular staining in the cytoplasm, membrane, or nucleus, which was interpreted as positive. Whole slide images were produced with a digital slide scanner PANORAMIC SCAN (3DHISTECH, Hungary), and all micrographs were exported by viewing whole slide images with SlideViewer version 2.7 (3DHISTECH).

**Table 1:** The information of IHC primary antibodies.

Antibody	Clone	Source	Manufacturer	Dilution	Staining pattern
GFAP	MX047	Mouse	Maxin	1:200	C
S-100	4C4.9	Rabbit	Maxin	1:1	C/N
OLIG2	ED112	Rabbit	Maxin	1:200	N
EMA	E29	Mouse	Maxin	1:1	C/M
Vimentin	MX034	Mouse	Maxin	1:200	C
Ki-67	MXR002	Mouse	Maxin	1:1	N

C/M indicates cytoplasmic/membranous staining; C/N, cytoplasmic/nuclear staining.

### Fluorescence *in situ* hybridization

EWSR1 and MN1 red and green two-color break-apart rearrangement probes (LBP medicine, China) were employed, each comprising two corresponding fluorescence in situ hybridization (FISH) DNA probes. The GSP EWSR1 gene 5′ red break probe spans chr22: 29,191,000–29,630,000, while the GSP EWSR1 gene 3′ green break probe spans chr22:29,679,000–30,609,000. Additionally, the GSP MN1 gene 5′ red break probe covers chr22:28,230,000–28,471,000, and the GSP MN1 gene 3′ green break probe spans chr22:27,746,000–28,120,000. Tissue pretreatment procedures were conducted in accordance with the provided instructions. Following the addition of the probe, the tissue was denatured at 85°C for 5 min and subsequently hybridized at 37°C overnight. After washing the slides the following day, DAPI was added dropwise, and the signals were observed under a fluorescence microscope (BX51, Olympus, Japan). A minimum of 100 non-overlapping interphase nuclei were analyzed. Among the identified broken genes, a fused yellow signal, a distinct green signal, and a separate red signal were observed within the nucleus. Furthermore, the presence of both a yellow fusion signal and either a separate red or green signal in the nucleus is indicative of gene rearrangement. When red and green separation signals are detected in more than 15% of tumor cell nuclei (with a separation diameter exceeding the combined diameters of the two signals), this is interpreted as a positive indication of EWSR1 or MN1 gene breakage.

### Next-generation sequencing and data processing

Routine paraffin-embedded tissue sections were enriched for areas of tumour cells (>70%) based on HE staining and tissue DNA was extracted according to the instructions of the QIAamp DNA FFPE Tissue kit (Qiagen, Hilden, Germany). DNA was then quantified using a Qubit fluorometer using Qubit dsDNA BR (Life Technologies, Carlsbad, CA). Qubit utilizes fluorescent dyes to selectively bind to specific target molecules. Upon binding to the DNA in the sample, the fluorescent dye emits a signal, which allows for the quantification of the DNA associated with the dye. The procedure involves adding 1 μL of the sample to be tested to a mixture of Qubit^TM^ dsDNA HS Reagent and Qubit^TM^ dsDNA buffer at a ratio of 1:199. The mixture is then vortexed to ensure thorough mixing before being placed into the Qubit fluorometer for detection. Next-generation sequencing was performed based on the Illumina NovaSeq 6000 next-generation sequencing platform using the SimcereDx NeuroOnco 360 NinAnco^™^ Brain Tumor Precision Diagnosis and Treatment Panorama Edition panel (Nanjing Simcere Diagnostics Co, Ltd., China). Three cases (cases 1, 3, and 4) underwent validation through the Illumina NextSeq 500-based RNA-targeted next-generation sequencing assay platform. For further validation, Two cases (cases 1 and 3) were subjected to fusion gene analysis of intron, promoter, or fusion breakpoint regions of 649 genes in RNA of tumour tissues at the Department of Molecular Diagnostics, Sun Yat-sen University Cancer Center using the DNA + RNA cancer-related gene panel (OrigiMed, Shanghai, China). Case 4 underwent fusion gene analysis of 649 genes in the RNA of tumour tissues at Nanjing Geneseeq Technology Inc. for 535 genes panoramic detection of central nervous system tumours and central metastatic tumours, covering point mutations, insertions/deletions, fusions, amplifications, chromosomal variations, methylation, and other variant types. Additionally, case 2 was submitted for DNA methylation profiling by Guangzhou Huayin Medical Laboratory Center. The sequencing library preparation and data analysis methods for next-generation sequencing were based on previously published literature (Cao et al., 2022; Zhang et al., 2023; Tan et al., 2024; Xi et al., 2024). Genomic DNA or double-stranded cDNA obtained through reverse transcription is fragmented, followed by end repair, ligation, PCR enrichment, and other steps to prepare the library. This library is then added to a sequencing chip, amplified via PCR, and subjected to high-throughput sequencing using fluorescence-labelled deoxyribonucleoside triphosphate. The resulting data is filtered and bioinformatically analyzed using a standardized automated sample management and data analysis system for raw data.

## Results

### Samples and clinical data

Detailed clinical information for each patient is provided in Table 2. The study included four female patients with a mean age of 24.75 years (range 8–44 years). Disease onset occurred in the spinal cord for three patients and in the right parietal area for one patient. Symptoms were specific to the site of onset and included weakness in the extremities, difficulty walking, and headaches with blurred vision.

**Table 2:** Clinical information on patients with astroblastoma in this study.

Patient	Age (years)	Sex	Location	Histologic features	Fusion	Grade	Follow-up (months)
Case 1	19	Female	Spinal cord	Astroblastoma	EWSR1-BEND2	High	76.5, alive
Case 2	8	Female	Spinal cord	Astroblastoma	EWSR1-BEND2	High	17.6, alive
Case 3	44	Female	Right parietal	Astroblastoma	EWSR1-NUDT10	High	33.7, alive
Case 4	28	Female	Spinal cord	Astroblastoma	MN1-BEND2	High	61.3, alive

### Pathological features

**Figure 1:**
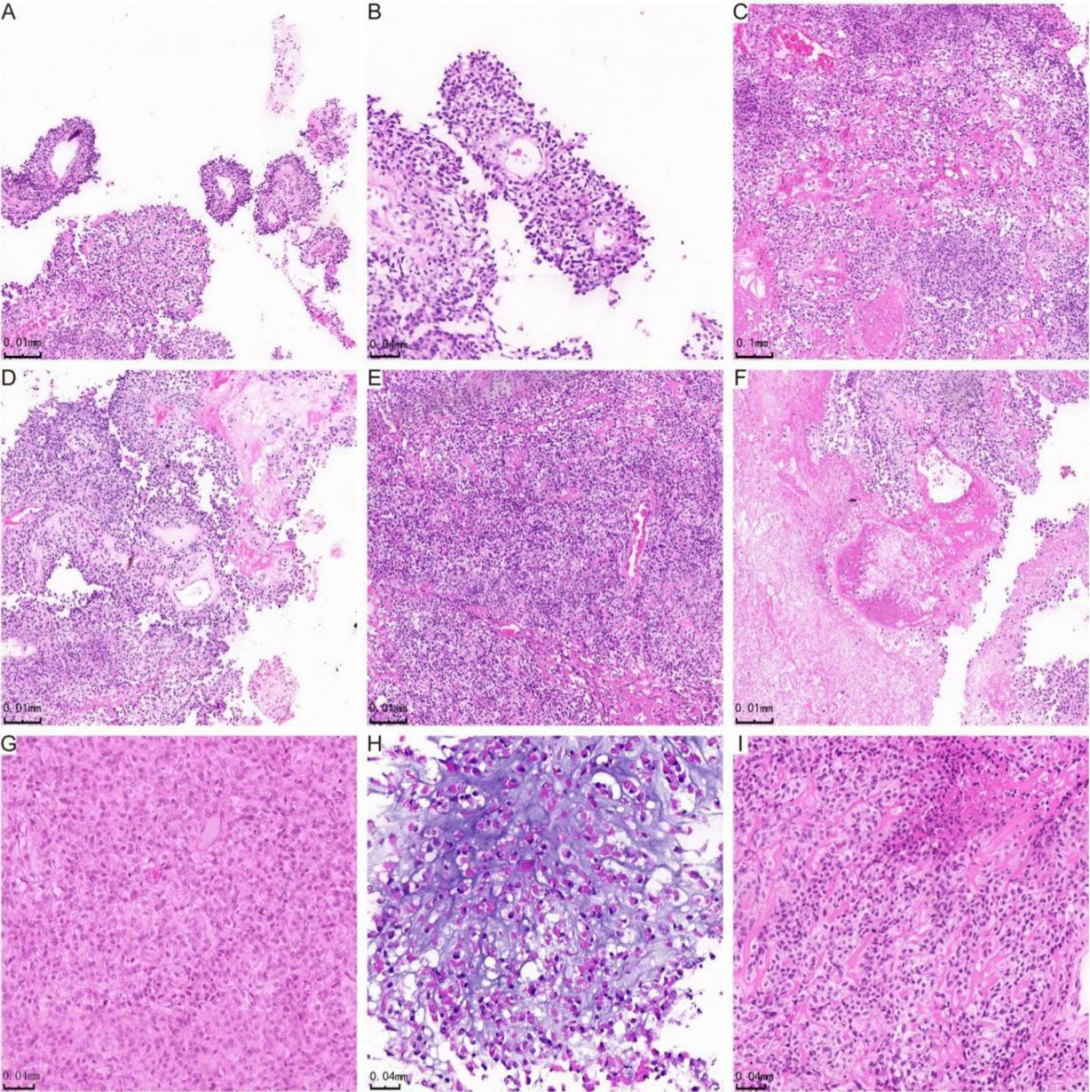
H&E staining showing histological manifestations of astroblastoma. **(A)** The tumor cells are organized in false rosette-like clusters surrounding blood vessels and exhibit a papillary growth pattern. **(B)** Shows the tumor cells arranged in a radial pattern. **(C)** The cells of the astroblastoma are positioned within a hardened matrix. **(D)** Highlights flake and trabecular structures, along with abundant vascularity and perivascularity. **(E)** Demonstrates prominent hyaline degeneration and a high density of tumor cells. **(F)** Indicates areas of necrosis, while **(G)** reveals undifferentiated cells characterized by abundant eosinophilic cytoplasm. **(H)** Star-shaped microcystic structures are observed in the astroblastoma, and **(I)** illustrates cells in astroblastomas exhibiting an epithelioid morphology.

Histologically, astroblastoma is characterized by tumor cells arranged in pseudorosette-like clusters or clumps around blood vessels, forming either solid or loose papillary structures (Figure 1A). The cell processes are thickened and radiate towards the central vessels (Figure 1B). In certain regions, cells are organized in sheets and trabeculae within a sclerotic matrix (Figure 1C). Abundant blood vessels and significant perivascular hyaline degeneration are also observed (Figure 1D). Four cases were classified as high histological grade due to elevated tumor cell density (Figure 1E) and necrosis, including extensive necrosis in one case (Figure 1F). The tumors exhibited relatively undifferentiated cells with oval-shaped hyperchromatic nuclei and abundant eosinophilic cytoplasm (Figure 1G). One case presented areas of myxoid stroma and microcystic structures (Figure 1H). The tumor cells were uniform in size, and in addition to oval or round perivascular tumor cells, epithelioid cell morphology may also be present (Figure 1I). Figure 1H was derived from case 4, while the other images in Figure 1 were all from case 3.

### Immunohistochemistry results

**Figure 2:**
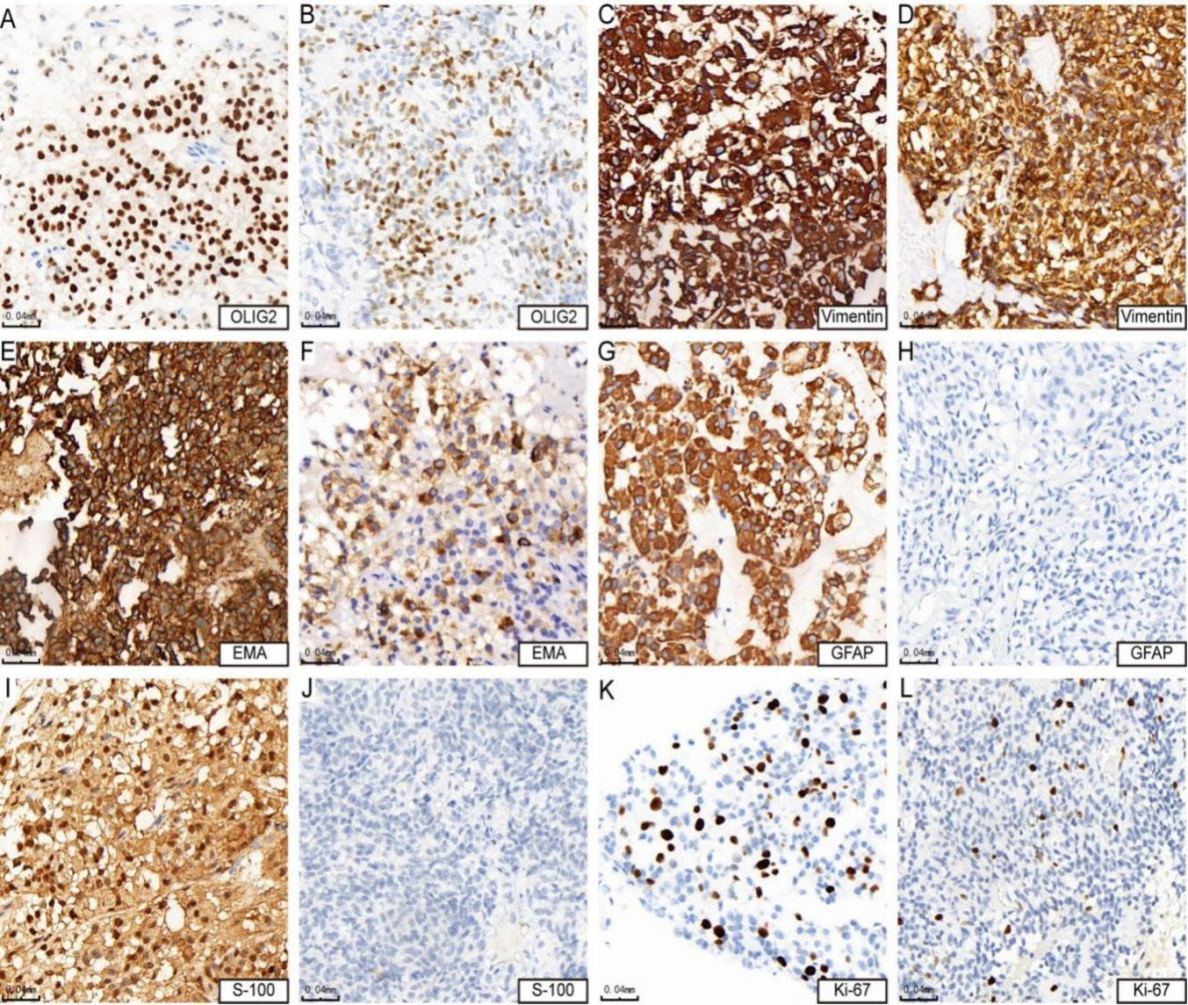
Immunohistochemical features of astroblastoma. **(A,B)** Show positive expression of OLIG2 (cases 3 and 4). **(C,D)** Demonstrate strong positive expression of vimentin (cases 3 and 4). **(E)** indicates strong positive expression of EMA (case 4), while **(F)** Shows positive expression of EMA (case 3). **(G)** Reveals strong positive expression of GFAP (case 4), whereas **(H)** shows negative expression of GFAP (case 3). **(I)** Presents positive expression of S-100 (case 4), in contrast to **(J)**, which indicates negative expression of S-100 (case 3). Lastly, **(K)** shows that the KI-67 hotspot area is approximately 15% + (case 4), while **(L)** indicates that the KI-67 hotspot area is about 10% + (case 3).

All cases expressed OLIG2 (4/4, Figures 2A,B), vimentin (Figures 2C,D), and EMA, with three cases showing strong positive expression (Figure 2E) and one case exhibiting focal expression (Figure 2F). Additionally, three out of four cases expressed GFAP (Figure 2G), while one case did not express GFAP (Figure 2H). Similarly, three cases (3/4) expressed S-100 (Figure 2I), and one case did not express S-100 (Figure 2J). The Ki-67 positive index was 15% in three cases (3/4) (Figure 2K) and 10% in one case (1/4) (Figure 2L). The results of immunohistochemical marker staining for the four samples are summarized in Table 3.

**Table 3:** IHC characteristics of astroblastoma cases.

Patient	GFAP	S-100	OLIG2	EMA	Vimentin	Ki-67
Case 1	+	+	Patchy+	+	+	15%
Case 2	+	Patchy+	Focal+	+	+	15%
Case 3	−	−	Patchy+	+	+	10%
Case 4	+	+	+	Focal+	+	15%

### FISH results

**Figure 3:**
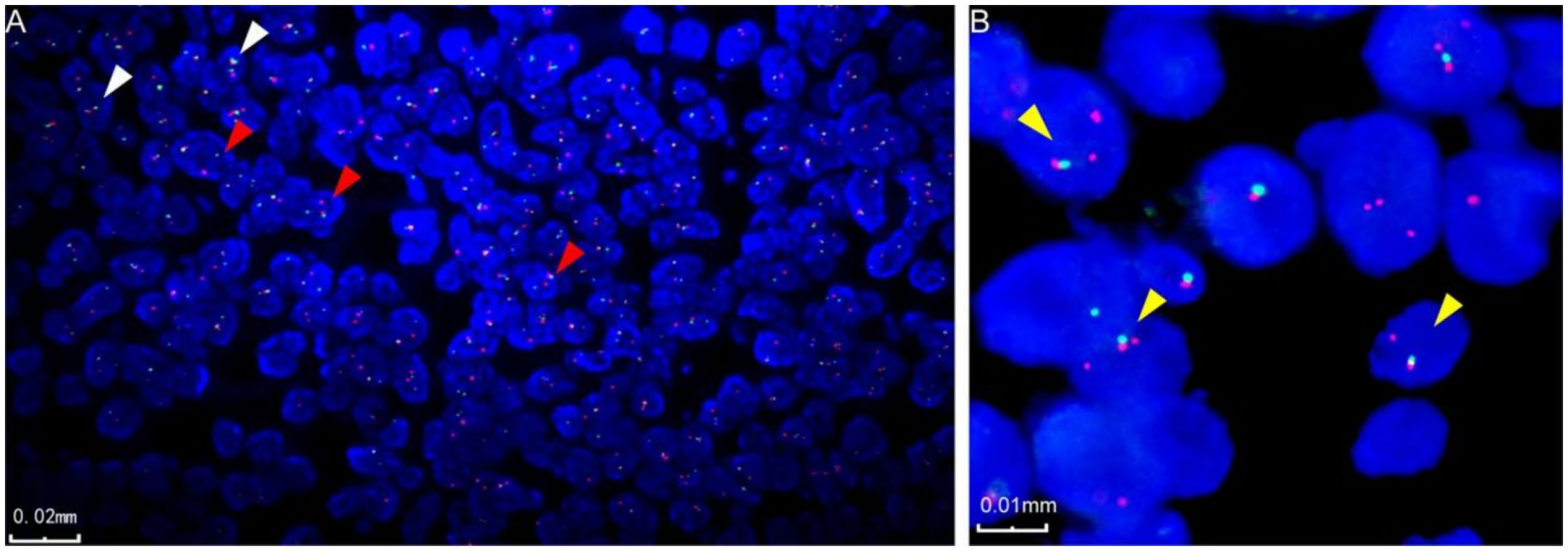
Fluorescence *in situ* hybridization characterization of astroblastoma. **(A)** Fluorescence in situ hybridization revealed EWSR1 gene disruption, indicated by the red arrow (case 3). In the negative case, two fused signals, red and green, are displayed in the image, as indicated by the white arrow (case 3). **(B)** Fluorescence in situ hybridization also demonstrated disruption of the MN1 gene, marked by the yellow arrow (case 4).

Two cases underwent detection using EWSR1 and MN1 fragmentation separation probes. In case 3 microscopy, 50% of the tumor cells exhibited separation of red and green signals, indicating fragmentation of the EWSR1 gene (Figure 3A). In case 4 microscopy, 70% of the tumor cells displayed isolated red signals along with a fusion signal, suggesting the presence of an MN1 gene break (Figure 3B).

### Next-generation sequencing results

**Figure 4:**
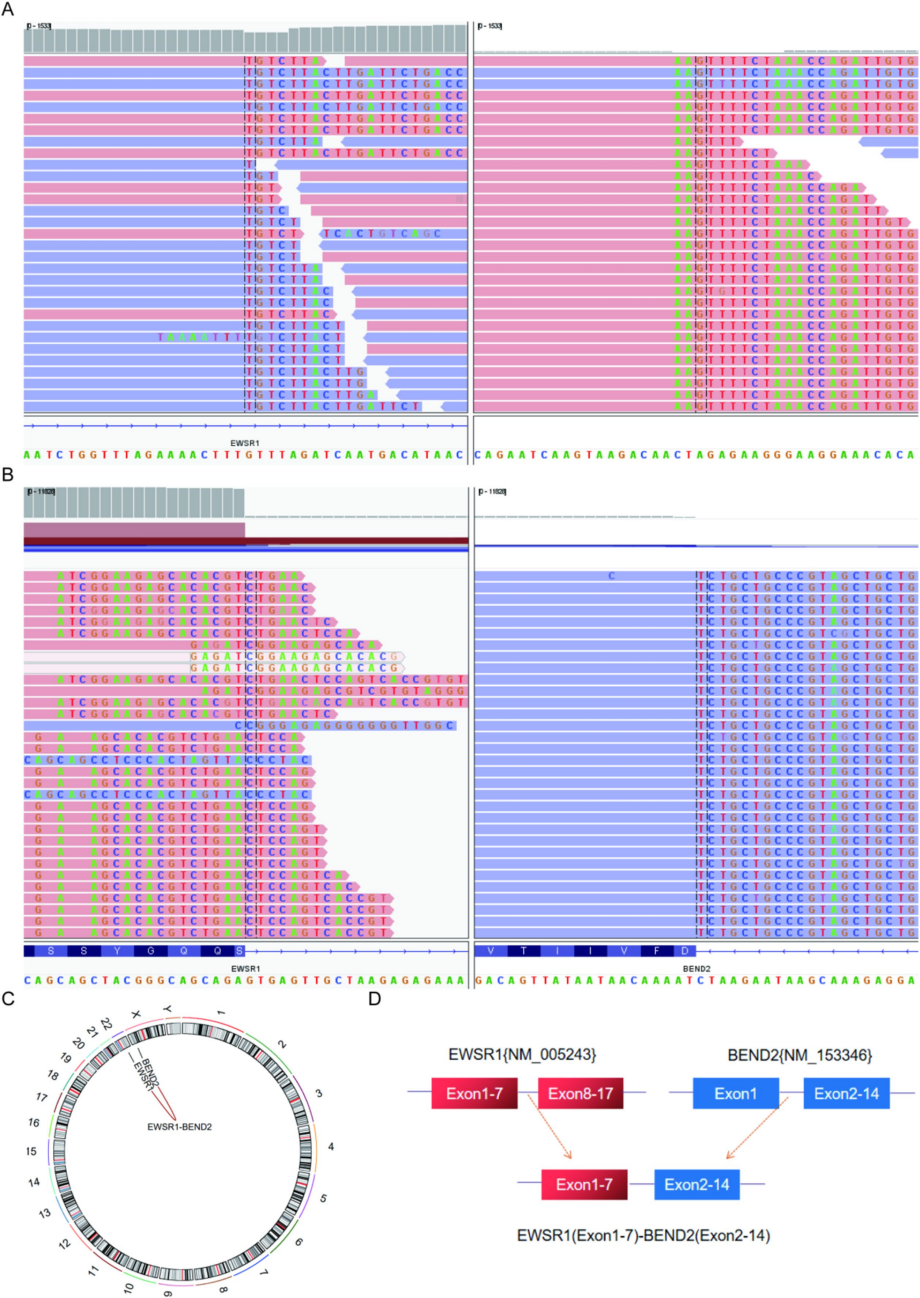
Molecular analysis of astroblastoma by next-generation sequencing. **(A)** EWSR1 (NM_005243:Exon1-7)-BEND2 (NM_153346:Exon2-14) fusion from case 1 in Integrated Genome Viewer (IGV). IGV screenshot showing breakpoints in exons 1–7 of the EWSR1 gene (left) and exons 2–14 of the BEND2 gene (right) detected by capture-based next-generation sequencing. **(B)** RNA sequencing shows EWSR1 (NM_005243:Exon1-7)-BEND2 (NM_153346:Exon2-14) fusion. **(C)** Results of chromosomal rearrangement involving EWSR1 (chr22:29685445) and BEND2 (chrX:18248771). Chromosomal rearrangement results. **(D)** Illustration of EWSR1-BEND2 fusion in case 1 based on next-generation sequencing results.

**Figure 5:**
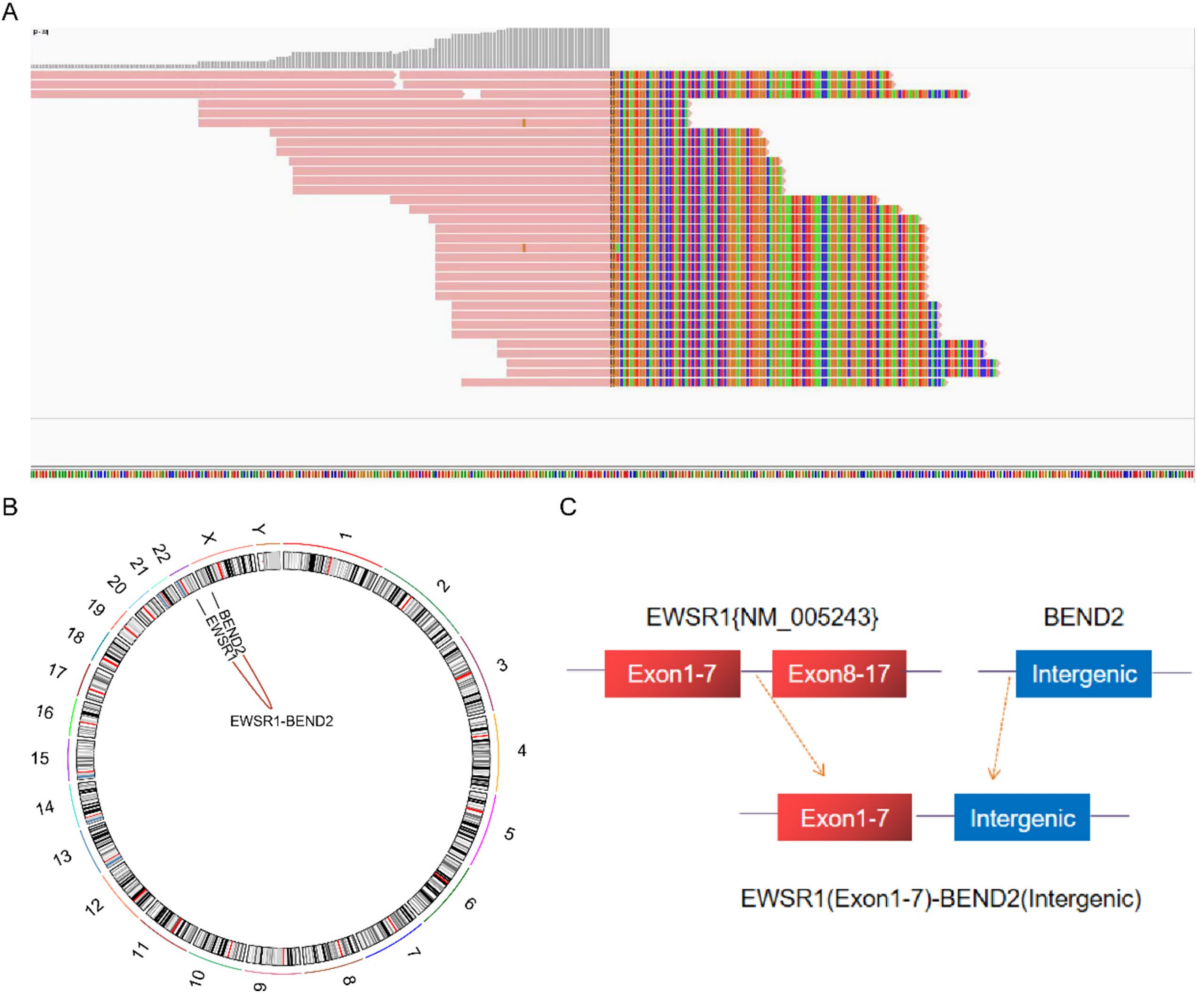
Molecular analysis of astroblastoma by next-generation sequencing. **(A)** EWSR1 (NM_005243:Exon1-7)-BEND2 (Intergenic) fusion from case 2 in Integrated Genome Viewer (IGV). IGV screenshot showing breakpoints on exons 1–7 of the EWSR1 gene (left) and the BEND2 intergenic region (right) detected by capture-based next-generation sequencing. **(B)** Results of chromosomal rearrangement involving the interregion genes EWSR1 (chr22:29685057) and BEND2 (chrX:18238601). **(C)** Illustration of EWSR1-BEND2 fusion in case 2 based on next-generation sequencing results.

**Figure 6:**
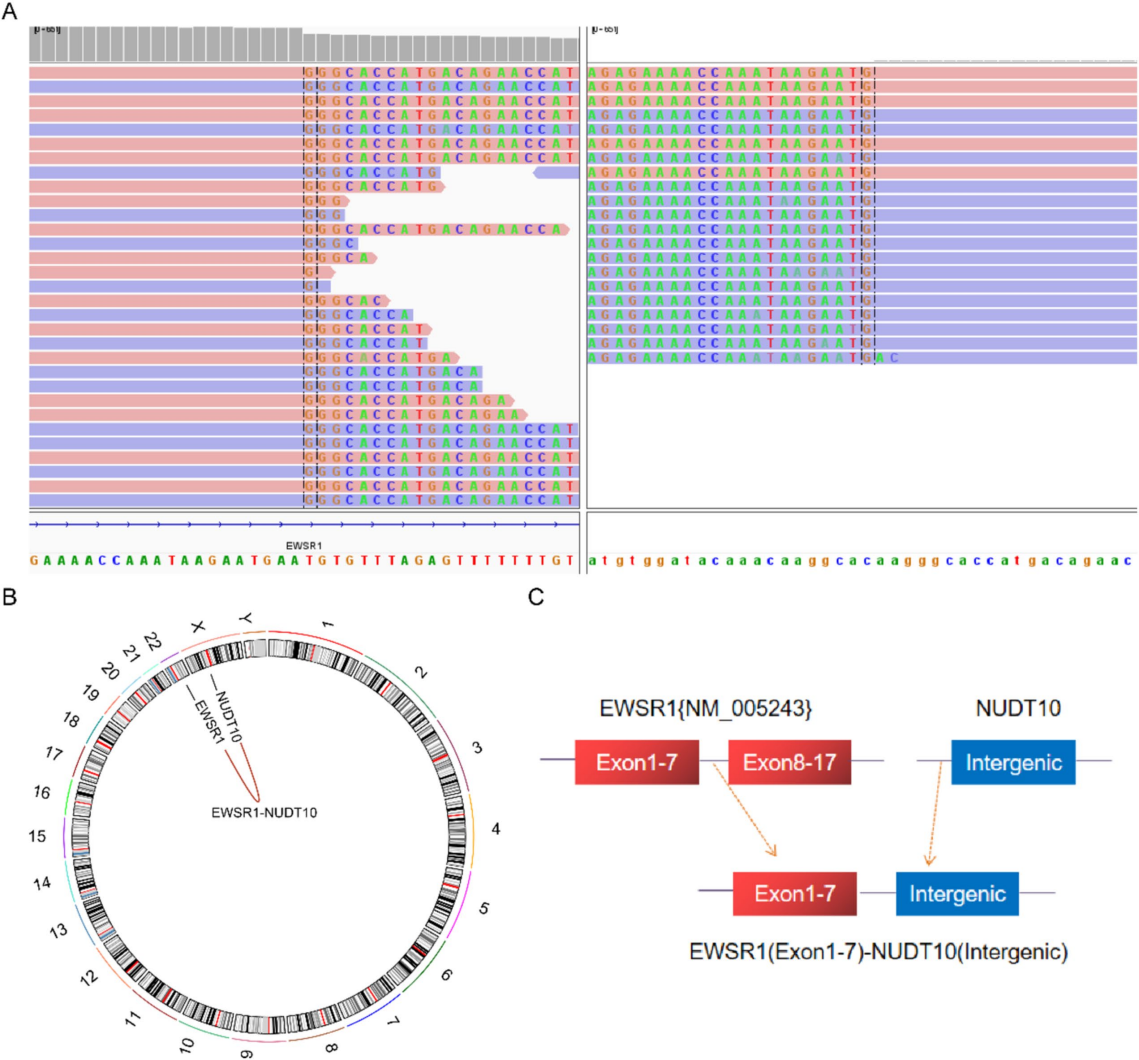
Molecular analysis of astroblastoma by next-generation sequencing. **(A)** EWSR1 (NM_005243:Exon1-7)-NUDT10(Intergenic) fusion from case 3 in Integrated Genome Viewer (IGV). IGV screenshot showing breakpoints on exons 1–7 of the EWSR1 gene (left) and the NUDT10 intergenic region (right) detected by capture-based next-generation sequencing. **(B)** Results of chromosomal rearrangements involving the interregion genes EWSR1 (chr22:29683160) and NUDT10 (chrX:51098723). **(C)** Illustration of EWSR1-NUDT10 fusion in case 3 based on next-generation sequencing results.

**Figure 7:**
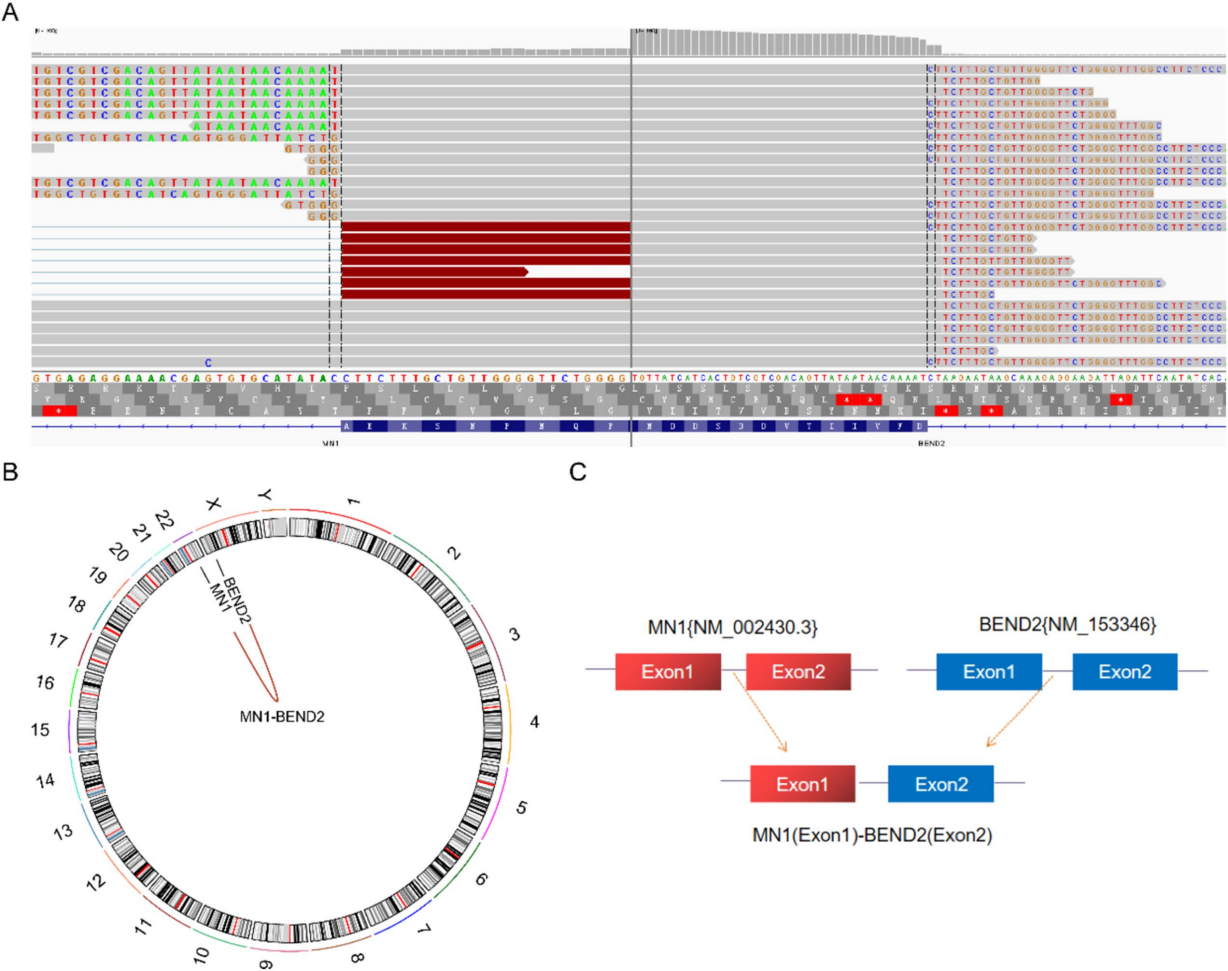
Molecular analysis of astroblastoma by next-generation sequencing. **(A)** MN1 (NM_002430.3:Exon1)-BEND2 (NM_153346.5:Exon2) fusion from case 4 in Integrated Genome Viewer (IGV). IGV screenshot showing breakpoints on MN1 gene exon 1 (left) and BEND2 gene exon 2 (right) detected by capture-based next-generation sequencing. **(B)** Results of chromosomal rearrangements involving the MN1 (chr22:28192751) and BEND2 (chrX:18234853) genes. Chromosomal rearrangement results. **(C)** Illustration of MN1-BEND2 fusion in case 4 based on next-generation sequencing results.

Next-generation sequencing results revealed the presence of a fusion between the EWSR1 gene and the BEND2 gene in case 1 (Figure 4A). RNA verification results (Figure 4B) indicated that the chromosomal rearrangement occurred at the following positions: EWSR1 (chr22:29685445) and BEND2 (chrX:18248771), resulting in the EWSR1-BEND2 fusion (Figure 4C). This gene fusion encompasses exons 1 to 7 of the EWSR1 gene (NM_005243) and exons 2 to 14 of the BEND2 gene (NM_153346) (Figure 4D). In case 2, sequencing also identified the EWSR1-BEND2 fusion (Figure 5A), with the chromosomal rearrangement occurring between the EWSR1 (chr22:29685057) and BEND2 (chrX:18238601) regions. This fusion (Figure 5B) involves exons 1 to 7 of the EWSR1 gene (NM_005243) and the intergenic region of the BEND2 gene (Figure 5C). In case 3, DNA detection revealed a fusion between the EWSR1 gene and the NUDT10 intergenic region (Figure 6A), with the chromosomal rearrangement occurring at the EWSR1-EWSR1 (chrX:51098723) intergenic region. The NUDT10 fusion (Figure 6B) involves the combination of exons 1 to 7 of the EWSR1 gene (NM_005243) and the NUDT10 intergenic region (Figure 6C). In case 4, RNA analysis detected a fusion of the MN1 gene and the BEND2 gene (Figure 7A), with the chromosomal rearrangement occurring at the MN1-BEND2 fusion of the MN1 (chr22:28192751) and BEND2 (chrX:18234853) genes (Figure 7B). This gene fusion involves the merging of MN1 gene exon 1 (NM_002430.3) and BEND2 gene exon 2 (NM_153346.5) (Figure 7C). Additionally, case 2 was classified as astroblastoma based on DNA methylation profiling.

### Prognosis

**Figure 8:**
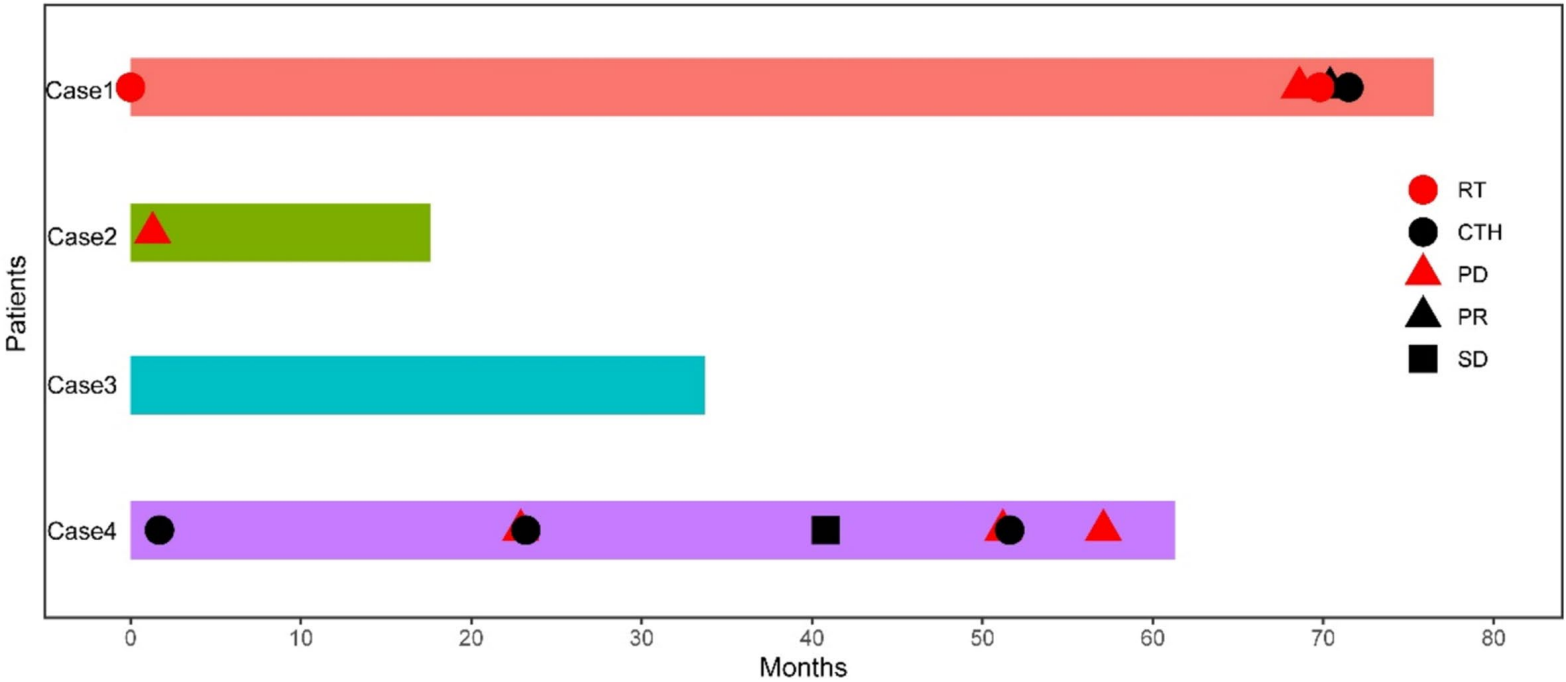
Swimming chart showing the clinical treatment characteristics of four patients with astroblastoma. RT, radiotherapy; CTH, chemotherapy; PD, progressive disease; PR, partial response; SD, stable disease.

The follow-up period for case 1 was 76.5 months. After surgery at the local hospital, radiotherapy was conducted, with recurrence happening 68.6 months later. Subsequent resection of the recurrent tumour was followed by another round of radiotherapy. An MRI review 1.2 months later indicated a partial response (PR) of the tumour. The patient continued chemotherapy and is currently alive. Case 2 had a follow-up period of 17.6 months, with no postoperative radiotherapy or chemotherapy. Despite the relapse occurring 1.3 months later, the patient is still alive. Case 3 has been followed up for 33.7 months, without receiving radiotherapy or chemotherapy post-surgery, and has not experienced recurrence or metastasis, remaining alive to this day. In case 4, the follow-up period was 61.3 months. Postoperative chemotherapy was administered at the local hospital, with recurrence happening 22.9 months later. The recurrent tumour was resected, and a combination of chemotherapy and radiotherapy was again performed locally. An MRI review 17.7 months later showed stable disease (SD) status of the tumour. After 28 months, a second recurrence occurred, leading to surgical removal of the recurrent tumour followed by chemotherapy. A third recurrence happened 5.5 months later. Clinicians determined that the patient was not fit for another surgery, the patient is currently alive without any further treatments (Figure 8).

## Discussion

The fifth edition of the WHO classification of central nervous system tumors recognizes molecular genetic alterations as a critical parameter for tumor updating, classification, typing, and grading. In primary central nervous system tumors, changes in the MN1 gene are uncommon and typically associated with MN1 translocations. Neuroepithelial tumors are even rarer, exhibiting diverse histomorphological features and biological behaviors, which complicates their diagnosis significantly. Advancements in molecular detection technology have facilitated the identification of an increasing number of tumor entities characterized by unique genetic changes. In the 2021 World Health Organization (WHO) classification of central nervous system tumors, astroblastoma is categorized as a localized astrocytic glioma and designated as “astroblastoma, MN1 variant.” The specific classification type remains unclear, and the WHO classification criteria for this variant have yet to be established (Louis et al., 2021).

Astroblastoma is a rare central nervous system tumour with limited current research and lacking accurate clinical epidemiological data. It can occur at any age but is most common in children and young individuals. Reports indicate a median age of diagnosis around 14 years, with a higher prevalence in women compared to men, roughly 8:1 to 11:1 (Singh et al., 2022; Fu et al., 2024). Clinical signs and symptoms vary based on tumour location and size, often presenting as increased intracranial pressure and local nerve loss. Common symptoms include headache, epilepsy, and vomiting (Mallick et al., 2017; Joshi et al., 2022). In our study, the four patients were aged 8, 19, 28, and 44, all female. The median age of onset in our study was 23.5 years, slightly higher than previous reports, with a similar gender distribution. Clinical symptoms were nonspecific, with headache and dizziness being the most common. Tumours were predominantly found in the superficial cortex of the supratentorial cerebral hemisphere, particularly in the frontal and parietal lobes, followed by the temporal and occipital lobes, and rarely in other locations such as the cerebellum, optic nerve, corpus callosum, brainstem, and spinal cord (Smith-Cohn et al., 2021; Rao et al., 2022). Among the four patients studied in our study, three patients had lesions located in the spinal cord and one patient located in the parietal lobe.

Astroblastoma typically presents histological characteristics such as tumour cells arranged in a pseudorosette or papillary shape around small blood vessels, with thick and short cytoplasmic protrusions near the central vessels, oval or round nuclei eccentrically positioned away from vessels, and hyaline degeneration in vessel walls. Scholars commonly classify astroblastomas into low-grade and high-grade based on features like cell arrangement, density, atypia, and mitotic figures. High-grade astroblastomas display multilayered cell proliferation around vessels, significant cell atypia, visible mitotic figures, endothelial cell proliferation, and the absence of hyaline degeneration in vessel walls (Shin et al., 2018; Chen et al., 2020). Despite these histologic features, the morphology can vary and may occasionally resemble other central nervous system tumors, such as ependymoma, thereby complicating the diagnostic process. Case 1 in this study was diagnosed with ependymoma at an early stage, while case 4 exhibited epithelioid features in histological morphology. Molecular genetic analysis through next-generation sequencing can aid pathologists in avoiding such diagnostic pitfalls.

Immunohistochemical staining typically shows positive results for characteristic markers like GFAP, vimentin, and S-100, although the level of positivity can vary. Interestingly, in our study, one case tested negative for GFAP and S-100 in immunohistochemical staining, aligning with findings reported by other researchers (Mastrangelo et al., 2010; Mellai et al., 2015). Limited cases like these and incomplete molecular research have led to speculation in the literature that this discrepancy may be attributed to the use of different antibodies. Notably, in our study, cases were analyzed using second-generation sequencing, revealing a novel fusion gene EWSR1-NUDT10. Despite receiving no adjuvant treatment, none of the patients experienced recurrence. It is hypothesized that cases negative for GFAP may exhibit different prognostic outcomes compared to GFAP-positive cases, a hypothesis that warrants further investigation through extensive data verification. All four patients in this study were diagnosed with high-grade astroblastoma, with Ki-67 positive index ranging from 10 to 15%. The relationship between Ki-67 index and prognosis remains unclear, highlighting the need for additional research.

Molecular genetic analysis of astroblastoma DNA or RNA using targeted next-generation sequencing reveals frequent MN1 mutations, often presenting as MN1-BEND2 fusion (Chen et al., 2020). However, mutations such as IDH, ATRX, and BRAF are absent (Ramkissoon et al., 2017; Hirose et al., 2018; Lehman et al., 2019). Recent studies have identified EWSR1-BEND2 fusion in astroblastoma, primarily located in the spinal cord or brain stem, categorized under MN1-altered astroblastoma methylation (Yamasaki et al., 2020).

Previous studies have identified EWSR1-BEND2 fusion in two cases of spinal cord astroblastoma (Yamasaki et al., 2020; Tsutsui et al., 2021), although the direct impact of this fusion on tumorigenesis remains unclear. Among the four patients in our study, three had EWSR1 fusion, one had EWSR1(Exon1-7)-BEND2(Exon2-14) fusion, one had EWSR1(Exon1-7)-BEND2 (Intergenic) fusion, and one had EWSR1(Exon1-7)-NUDT10(Intergenic) fusion. The EWSR1(Exon1-7)-NUDT10(Intergenic) fusion is a newly discovered fusion, indicating a novel chromosomal fusion pattern not previously observed in astroblastoma tumours. This fusion may play a key role in the pathogenesis of this tumour, although further investigation is needed to fully understand its functional implications. Targeted RNA sequencing was conducted to validate this case and confirm the presence of the fusion gene in the intergenic region. Unfortunately, due to the degraded quality of the sample, verification attempts were unsuccessful. Additionally, the fusion was associated with other alterations, including negative expression of GFAP and S-100. Considering the distinct clinicopathological and molecular features, it is suggested that this could represent a potential new subtype of astroblastoma characterized by EWSR1-NUDT10 fusion as a molecular hallmark. However, further data collection is required to support this hypothesis before drawing definitive conclusions. Our study is the first to demonstrate this rearrangement, the pathogenic and diagnostic implications of the EWSR1-NUDT10 fusion remain incompletely understood. The tumour examined in our study exhibited a favourable prognosis and no recurrence. Further research involving cases with similar molecular and clinicopathological features is necessary to elucidate the significance of this fusion. Prior literature has identified EWSR1-BEND2 fusions in pancreatic neuroendocrine tumours, spinal cord ependymoma, and tongue base adenocarcinoma (Scarpa et al., 2017; Todorovic et al., 2020; Agaimy et al., 2023). These EWSR1-BEND2 fusions involve the N-terminal activation domain of EWSR1 and the C-terminus of BEND2, emphasizing the essential role of both domains in tumorigenesis.

The MN1 gene, located on chromosome 22q12.1, spans approximately 53 Kb and consists of two exons that collectively encode 1,320 amino acids. Exon 1 encodes 1,260 amino acids, while exon 2 encodes the remaining 60 amino acids. Research has identified a co-regulatory gene of MN1 on chromosome 22, which is implicated in mutations associated with human brain tumours (Miyake et al., 2020). Notably, MN1 rearrangements are closely linked to astroblastoma, with a common genetic alteration being the MN1-BEND2 fusion. Burford et al. (2018) reported a recurrent case of MN1-BEND2 fusion, suggesting that MN1 disruption may play a causative role. Our study also found a recurrent MN1 (exon1)-BEND2 (exon2) fusion case. Additionally, the MN1-CXXC5 fusion gene is prevalent in astroblastoma, with BEND2 overexpression primarily observed in MN1-BEND2 tumours rather than MN1-CXXC5 tumours (Sturm et al., 2016). This indicates biological distinctions among tumours with different MN1 gene fusion partners. Furthermore, the MN1-BEND2 fusion gene has been identified in soft tissue sarcomas, suggesting its role as a pleiotropic fusion gene across various tumour types (Yoshida et al., 2022).

Astroblastoma shares morphological similarities with various tumours such as ependymomas, papillary meningiomas, papillary glial neuronal tumours, and choroid plexus papillomas. However, these tumours often present unique pathological or molecular features. Hyaline degeneration of blood vessel walls is rare or absent, pseudorosette tumour cells around blood vessels have slender cell processes and are negative for GFAP. Hyaline degeneration has a pseudopapillary structure around blood vessels, and there are no molecular changes in MN1 or EWSR1, which can be distinguished from it.

Due to the rarity of astroblastoma, there remains a lack of well-established treatment methods and prognosis guidance both domestically and internationally. Total surgical resection is currently the preferred treatment option, while the primary postoperative adjuvant treatment consists of combined radiotherapy and temozolomide (Lehman et al., 2022). It is commonly understood that low-grade astroblastoma typically presents as a slow-growing tumour with a favourable prognosis, although instances of recurrence have been documented in the literature (Fuller and Scheithauer, 2007; Sughrue et al., 2011). On the other hand, high-grade astroblastoma is more aggressive and has the potential to transform into glioblastoma-like tumours. Despite complete tumour removal, recurrence is common and the prognosis is often uncertain (Mastrangelo et al., 2010). In this study, all four patients had high-grade astroblastomas, with three experiencing recurrence post-surgery. The Ki-67 positive index ranged from 10 to 15%. The aggressive growth pattern of these tumors is believed to be the underlying cause of their recurrence. Factors influencing long-term survival include tumour location, the extent of surgical resection, and response to adjuvant therapy. Chen et al. (2020) conducted a meta-analysis involving 73 patients with astroblastoma cell tumours and MN1 variant variants, revealing a median progression-free survival (PFS) of 34 months and a median survival time of 184 months. Among the four patients in our study, three experienced recurrence, with the patient exhibiting MN1 fusion having multiple recurrences, consistent with existing literature. Notably, all four patients survived, with the longest survival period reaching 76.5 months.

## Conclusion

In summary, we present cases of MN1-BEND2 and EWSR1-BEND2 gene fusions in astroblastomas, alongside the first identification of novel EWSR1-NUDT10 fusions. This work contributes to the existing literature on astroblastomas associated with MN1 alterations. Further studies are required to delineate the molecular and clinicopathological spectrum of astroblastomas, which will enhance our understanding of their development and inform optimal treatment strategies for these rare tumors.

## Data Availability

The datasets presented in this study can be found in online repositories. The names of the repository/repositories and accession number(s) can be found in the article/supplementary material.
