# Corrigendum: Clinicopathological and molecular characterization of astroblastoma

**DOI:** 10.3389/fnmol.2025.1580624

**Published:** 2025-06-09

**Authors:** Xiaoyan Wu, Wenfeng Peng, Xu Zhang, Tao Tang, Ling Deng, Yuxia Xu, Xiaoyun Liu, Fang Wang, Wujian Peng, Jianrong Huang, Xiaoni Zhong

**Affiliations:** ^1^Department of Molecular Diagnostics, State Key Laboratory of Oncology in South China, Guangdong Provincial Clinical Research Center for Cancer, Sun Yat-sen University Cancer Center, Guangzhou, China; ^2^Department of Pathology, Shenzhen Second People's Hospital, Shenzhen University 1st Affiliated Hospital, Shenzhen University School of Medicine, Shenzhen, China; ^3^Department of Nephrology, The Third People's Hospital of Shenzhen, The Second Affiliated Hospital of Southern University of Science and Technology, Shenzhen, China; ^4^Department of Pathology, Shenzhen People's Hospital, The Second Affiliated Hospital of Jinan University, The First Affiliated Hospital of Southern University of Science and Technology, Shenzhen, China

**Keywords:** astroblastoma, clinicopathological, molecular characterization, next-generation sequencing, EWSR1-NUDT10 gene fusion

In the published article, the word “astrocytoma” was erroneously used in place of the correct word “astroblastoma” throughout the article including in the **title, keywords section, citation section, main body text, and the captions of** **Tables 2**, **3** **and** **Figures 1**, **2**. As astroblastoma is a rare and distinct CNS tumor that has been clearly established as an independent entity in the WHO classification system, the astroblastoma discussed in this research is therefore distinct from astrocytoma and should therefore be presented accurately. All mentions of astrocytoma have now been replaced with astroblastoma in the corrected article where appropriate. The corrected versions of Tables 2, 3 and Figures 1, 2, along with their revised captions, appear below.

**Table 2:** Clinical information on patients with astroblastoma in this study.

**Patient**	**Age (years)**	**Sex**	**Location**	**Histologic features**	**Fusion**	**Grade**	**Follow-up (months)**
Case 1	19	Female	Spinal cord	Astroblastoma	EWSR1-BEND2	High	76.5, alive
Case 2	8	Female	Spinal cord	Astroblastoma	EWSR1-BEND2	High	17.6, alive
Case 3	44	Female	Right parietal	Astroblastoma	EWSR1-NUDT10	High	33.7, alive
Case 4	28	Female	Spinal cord	Astroblastoma	MN1-BEND2	High	61.3, alive

**Table 3:** IHC characteristics of astroblastoma cases.

**Patient**	**GFAP**	**S-100**	**OLIG2**	**EMA**	**Vimentin**	**Ki-67**
Case 1	+	+	Patchy+	+	+	15%
Case 2	+	Patchy+	Focal+	+	+	15%
Case 3	−	−	Patchy+	+	+	10%
Case 4	+	+	+	Focal+	+	15%

**Figure 1:**
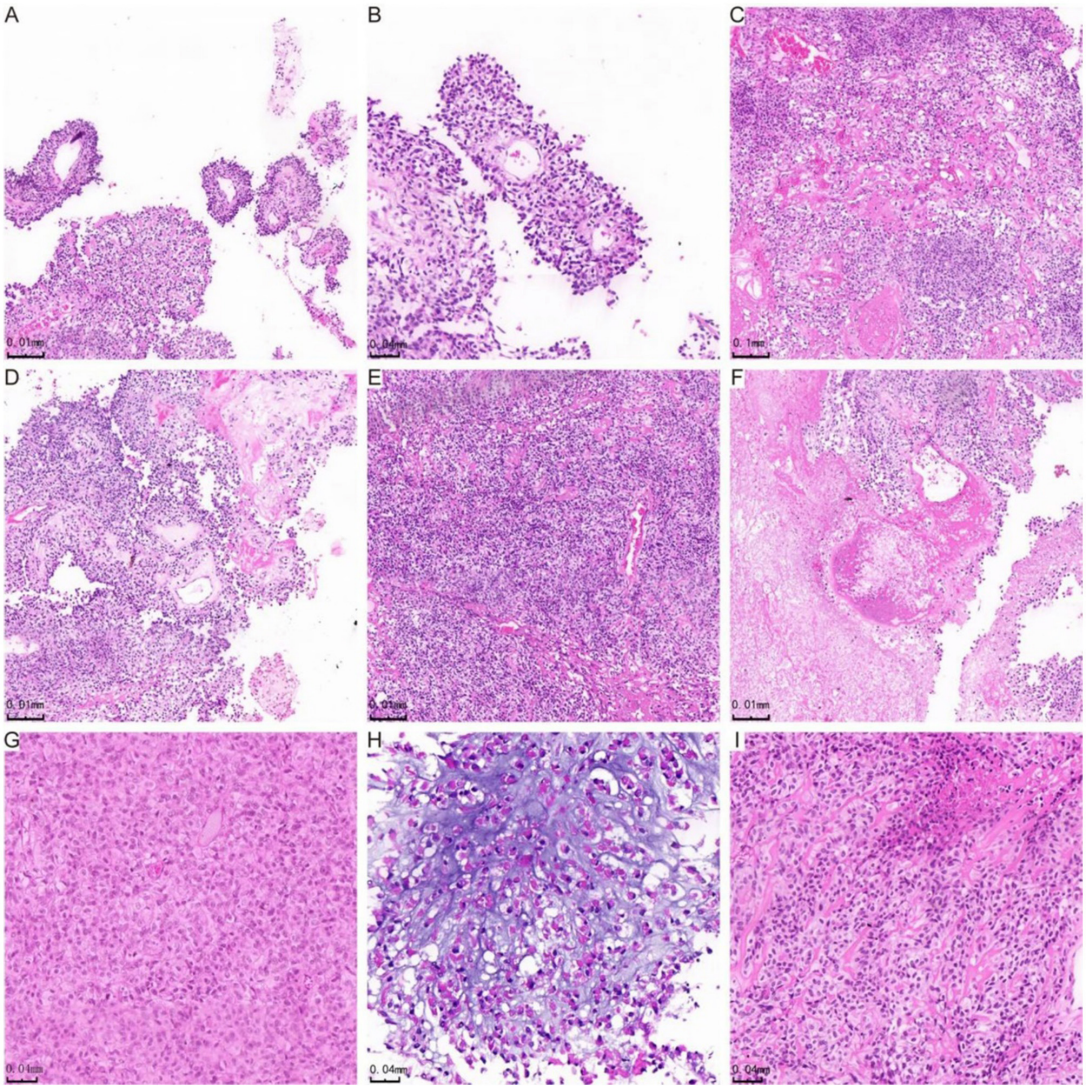
H&E staining showing histological manifestations of astroblastoma. **(A)** The tumor cells are organized in false rosette-like clusters surrounding blood vessels and exhibit a papillary growth pattern. **(B)** Shows the tumor cells arranged in a radial pattern. **(C)** The cells of the astroblastoma are positioned within a hardened matrix. **(D)** Highlights flake and trabecular structures, along with abundant vascularity and perivascularity. **(E)** Demonstrates prominent hyaline degeneration and a high density of tumor cells. **(F)** Indicates areas of necrosis, while **(G)** reveals undifferentiated cells characterized by abundant eosinophilic cytoplasm. **(H)** Star-shaped microcystic structures are observed in the astroblastoma, and **(I)** illustrates cells in astroblastomas exhibiting an epithelioid morphology.

**Figure 2:**
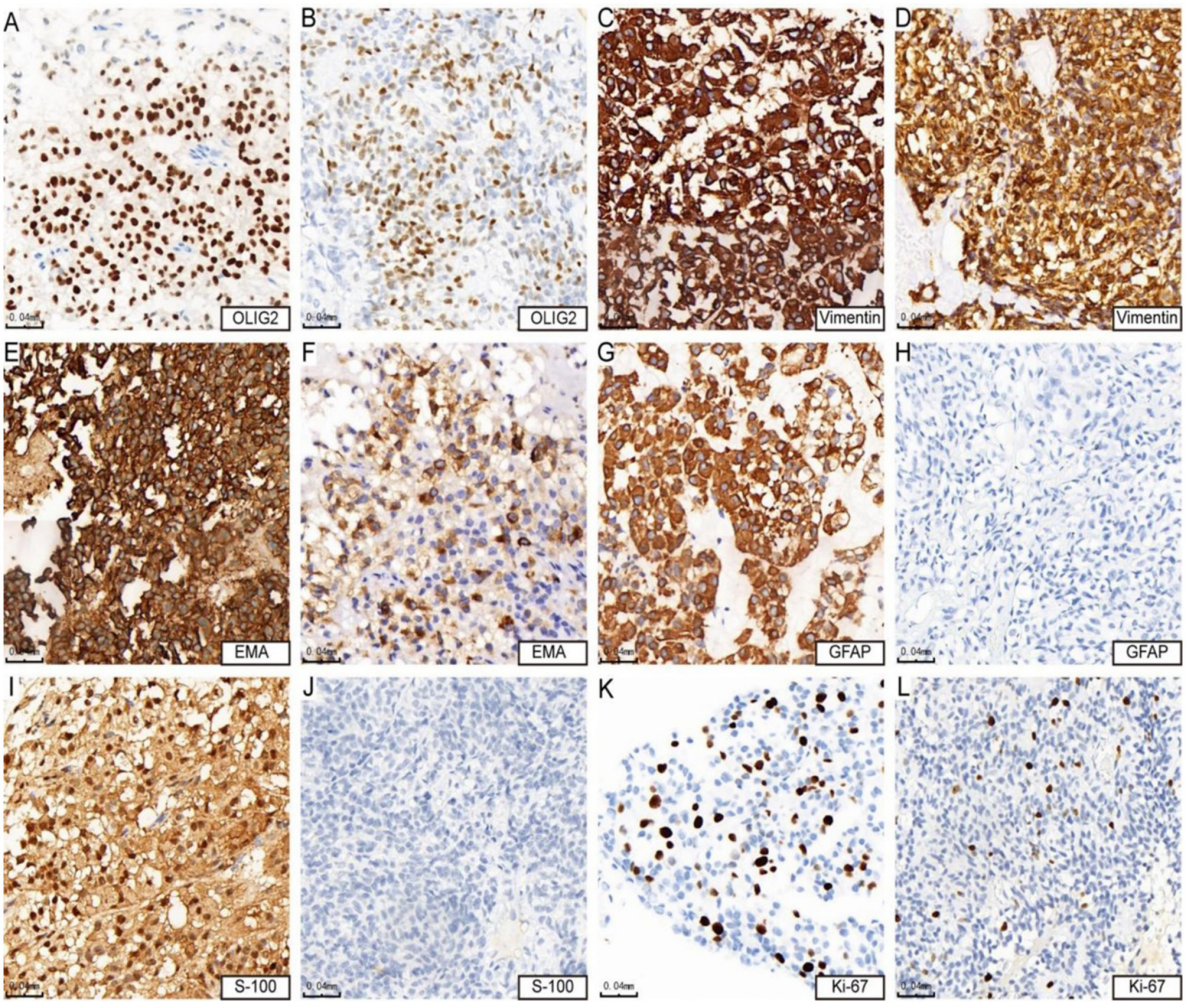
Immunohistochemical features of astroblastoma. **(A, B)** Show positive expression of OLIG2 (cases 3 and 4). **(C, D)** Demonstrate strong positive expression of vimentin (cases 3 and 4). **(E)** indicates strong positive expression of EMA (case 4), while **(F)** Shows positive expression of EMA (case 3). **(G)** Reveals strong positive expression of GFAP (case 4), whereas **(H)** shows negative expression of GFAP (case 3). **(I)** Presents positive expression of S-100 (case 4), in contrast to **(J)**, which indicates negative expression of S-100 (case 3). Lastly, **(K)** shows that the KI-67 hotspot area is approximately 15% + (case 4), while **(L)** indicates that the KI-67 hotspot area is about 10% + (case 3).

The authors apologize for this error and state that this does not change the scientific conclusions of the article in any way. The original article has been updated.

